# Cerebral hemodynamic characteristics of acute mountain sickness upon acute high-altitude exposure at 3,700 m in young Chinese men

**DOI:** 10.1007/s00421-014-2934-6

**Published:** 2014-07-05

**Authors:** Shi-Zhu Bian, Jun Jin, Qian-Ning Li, Jun Qin, Ji-Hang Zhang, Shi-Yong Yu, Jian-Fei Chen, Cai-Fa Tang, Lan Huang

**Affiliations:** 1 Department of Cardiology, Institute of Cardiovascular Diseases of PLA, Xinqiao Hospital, Third Military Medical University, Chongqing, 400037 China; 2 Department of Cardiology, Xinqiao Hospital, Third Military Medical University, Chongqing, China; 3 Department of Neurology, Xinqiao Hospital, Third Military Medical University, Chongqing, China

**Keywords:** Cerebral hemodynamic characteristics, Acute mountain sickness, High altitude

## Abstract

**Purpose:**

We aimed at identifying the cerebral hemodynamic characteristics of acute mountain sickness (AMS).

**Methods:**

Transcranial Doppler (TCD) sonography examinations were performed between 18 and 24 h after arrival at 3,700 m via plane from 500 m (*n* = 454). A subgroup of 151 subjects received TCD examinations at both altitudes.

**Results:**

The velocities of the middle cerebral artery, vertebral artery (VA) and basilar artery (BA) increased while the pulsatility indexes (PIs) and resistance indexes (RIs) decreased significantly (all *p* < 0.05). Velocities of BA were higher in AMS (AMS+) individuals when compared with non-AMS (AMS−) subjects (systolic velocity: 66 ± 12 vs. 69 ± 15 cm/s, diastolic velocity: 29 ± 7 vs. 31 ± 8 cm/s and mean velocity, 42 ± 9 vs. 44 ± 10 cm/s). AMS was characterized by higher diastolic velocity [*V*_d_VA_ (26 ± 4 vs. 25 ± 4, *p* = 0.013)] with lower PI and RI (both *p* = 0.004) in VA. Furthermore, the asymmetry index (AI) of VAs was significantly lower in the AMS + group [−5.7 % (21.0 %) vs. −2.5 % (17.8 %), *p* = 0.016]. The AMS score was closely correlated with the hemodynamic parameters of BA and the *V*_d_VA_, PI, RI and AI of VA.

**Conclusion:**

AMS is associated with alterations in cerebral hemodynamics in the posterior circulation rather than the anterior one, and is characterized by higher blood velocity with lower resistance. In addition, the asymmetry of VAs may be involved in AMS.

## Introduction

Acute mountain sickness (AMS) occurs temporarily in the individuals who ascend to 2,500 m or above. Lake Louise self-assessment scoring system (LLS) has been applied in its diagnosis, i.e., whoever arrives at high altitude experiences headache and has LLS score >3 (Imray et al. 2010). AMS has been considered as insufficiency of adaptation to high altitude and neurological disorders caused by hypoxia. Headache is the primary symptom according to LLS and is defined as high-altitude headache (HAH), if it occurs within 24 h after person ascents to 2,500 m or above and subsides within 8 h after descending (Carod-Artal 2012; Lawley 2011; Serrano-Duenas 2005). Thus, it is urgent and critical to identify the clinical characteristics of AMS due to its potential hazard for workers, mountaineers and tourists after acute high-altitude exposure.

Although AMS has been studied for many years, its underlying pathophysiological mechanisms of AMS are still not fully understood, including whether the ventilation response, circulatory alteration and the changes in cerebral blood flow (CBF) are involved. Specifically, alterations in CBF, brain volume and intracranial pressure may play crucial roles in the development of AMS, particularly the neurological symptoms, headache and dizziness (Imray et al. 2010). Modification in CBF due to high altitude is also a compensatory reaction that may be caused by vasoconstriction, elevated blood pressure (BP) and heart rate and the consequential rise in cardiac output (CO) to maintain sufficient supply of oxygen (or oxygen delivery) and energy for the brain. Initial constriction of cerebral vessels was induced by hypomania and subsequent dilation was caused hypoxia per se and the hypoxia-induced vasodilators (Brugniaux et al. 2007). CBF and the cerebral perfusion rise to a greater extent than what is reflected by the velocities of the cerebral vessels (Lassen 1992).

Although it has been indicated that CBF initially increases rapidly and is recovered progressively to what is at the sea level in 2 weeks, which is slower in high-altitude climbers as compared to the matched control group but without statistical significance (Rootwelt et al. 1986). However, the controversy result that CBF increases with acute hypoxia has been reported (Lassen 1992). Although a depression chamber study of 10 subjects has showed that AMS is not related to CBF (Baumgartner et al. 1999), CBF velocity responses to hypoxia in subjects who are susceptible to high-altitude pulmonary edema have been revealed (Berre et al. 1999).

Of note, most of these previous studies were conducted with small population sizes and based on simulations or only focused on the velocity of the middle cerebral artery (MCA) (Berre et al. 1999; Lucas et al. 2011; Rootwelt et al. 1986). Furthermore, the velocity of the unilateral MCA was applied as an evaluation or substitute for CBF, and the asymmetry of the bilateral cerebrovascular velocities has not been considered. Although one study did measure the mean blood flow velocities of both MCAs, only 23 subjects were recruited in the study (Baumgartner et al. 1994). On the other hand, HAH and dizziness in AMS may also be related to the posterior lobe of the brain that is supplied by the basilar artery (BA) and the vertebrobasilar arteries. Thus, we designed the present study to investigate the relationship between CBF [velocities of BA, bilateral MCAs and vertebral arteries (VAs)] and AMS, and thus to comprehensively identify the cerebral hemodynamic characteristics of AMS after acute ascent to high altitude.

## Methods

### Participants and procedures

#### Participants

Altogether, 454 subjects participated in the study according to the inclusion and exclusion criteria. The inclusion criteria were defined as that healthy male between 18 and 60 years. The exclusion criteria were as follows: people with any of the following conditions—respiratory system disease, cardiovascular system disease, neuropsychosis, cerebrovascular disease, malignant tumors or dysfunctions of the liver or the kidneys.

Subjects who agreed to participate in the study were fully familiar with the purposes and processes of this study and signed informed consents before examination. The study was approved by the Ethics Committee of Xinqiao Hospital at the Second Clinic Medical College of the Third Military Medical University.

#### Procedures

The subjects were recruited in June 2012. The field trials were performed between 18 and 24 h after their arrivals at 3,700 m by plane within 2 h in June 2012. The baselines were examined at 500 m within 1 week prior to their departures.

Structured case report form questionnaires were used to record demographic data (age, BMI, smoking and alcohol consumption) and the symptoms of AMS (headache, dizziness, gastrointestinal symptoms, difficulty sleeping and fatigue/weakness). The symptoms were scored as following: headache (0 = without headache; 1 = mild headache; 2 = moderate headache; 3 = severe headache), dizziness (0 = without dizziness; 1 = mild dizziness; 2 = moderate dizziness; 3 = severe dizziness), gastrointestinal symptom (0 = without and 1 = with gastrointestinal symptom), insomnia (0 = as well as usual; 1 = not so well as usual; 2 = wake up for times over the night and 3 = difficult to sleep) and fatigue (0 = without fatigue and 1 = with fatigue). AMS was diagnosed based on Lake Louise self-assessment scoring system (LLS): people who arrived at high altitude had headache and LLS score >3. Transcranial Doppler (TCD) sonography examinations were performed using an ultrasonography system with a 2 Hz probe (EME TC2021-III, NICOLET, USA) by the same technologist. After resting for 30 min, the subjects were placed in the supine position and then in the prone position to receive TCD examinations of the different cerebral arteries. The velocities (systolic velocity, diastolic velocity and mean velocity), pulsatility index (PI) and resistance index (RI) of the bilateral MCAs were measured via the temporal window (depth: 52 mm), and the rates of the bilateral VAs and BA were measured via the occipital window (depth: 60 and 76 mm). Those parameters were averaged in three cardiac cycles (set and calculated in the TCD system) after 1 min stability. The PI = (systolic velocity − diastolic velocity)/mean velocity and RI = (systolic velocity − diastolic velocity)/systolic velocity. Furthermore, both of pulsatility and resistance indexes were detected by using the TCD system. The BP was measured by sphygmomanometer (HEM-6200, OMRON, China). The systolic pressure (SBP) and diastolic pressure (DBP) and mean arterial pressure (MAP) have been recorded. Cerebrovascular conductance index (CVCi) = *V*_m_MCA_/MAP.

A subgroup of 151 volunteers received TCD and BP examinations at both 500 and 3,700 m.

### Statistical analysis

The case report forms were excluded if the demographic information was incomplete or the velocity could not be detected at the depths mentioned above. Totally, 18 case report forms were excluded.

Means of systolic velocity, diastolic velocity, mean velocity, RI and PI in the bilateral MCAs and VAs at 3,700 m were calculated as *V*_s_MCA_, *V*_d_MCA_, *V*_m_MCA_, *V*_s_VA_, *V*_d_VA_, *V*_m_VA_ RI_MCA_, RI_VA_, PI_MCA_, and PI_VA_, respectively. The normally distributed measurement variables (age, BMI, velocities of the BA, and means of the velocities of the bilateral MCAs and VAs) were expressed as the mean ± standard deviation (SD), while the non-normally distributed variables were presented as median (interquartile range). The enumerated data were expressed as rate of occurrence (%). The asymmetry of the MCA and VA was calculated as Δ*V* = left velocity − right velocity in each lateral MCA or VA, while the asymmetry index (AI) was calculated as AI = Δ*V*/[(left mean velocity + right mean velocity)/2]. *V*_s_MCA_, *V*_d_MCA_, *V*_m_MCA_, RI_MCA_, PI_MCA_, *V*_s_VA_, *V*_d_VA_, *V*_m_VA_ *V*_s_BA_, *V*_d_BA_, *V*_m_BA_, RI_MCA_, PI_MCA_, RI_VA_ and PI_VA_ were compared using paired *t* tests between the 500 and 3,700 m altitudes. The variables mentioned above were analyzed using independent samples *t* tests to make comparison between AMS+ and AMS− groups at 3,700 m, while the asymmetries and AIs were compared using Mann–Whitney *U* tests. The relationship between the AMS score and the above-mentioned parameters at 3,700 m was analyzed using Spearman’s correlation. The statistical analyses were performed by using SPSS 19.0 software for Windows. *p* ≤ 0.05 was considered to be statistically significant.

Statisticians from the Third Military Medical University were consulted for all of the statistical methods and results.

## Results

Altogether, 454 valid case report forms were collected, while 436 valid TCD examinations were performed at 3,700 m elevation. Among these measurements, baselines at 500 m were performed among 151 individuals. The mean age of the subjects in this study was 23.42 ± 4.39 years (mean ± SD), and the BMI was 21.73 ± 2.89 kg/m^2^. Percentages of smoking and alcohol drinking were 22.4 % (98 of 436) and 57.1 % (270 of 436), respectively. The ethnicity of the population was primarily Han Chinese (81.9 %). Overall, in this study, the incidences of HAH, dizziness and AMS upon acute exposure to 3,700 m were 74.3, 71.8, and 62.8 %, respectively.

**Fig. 1:**
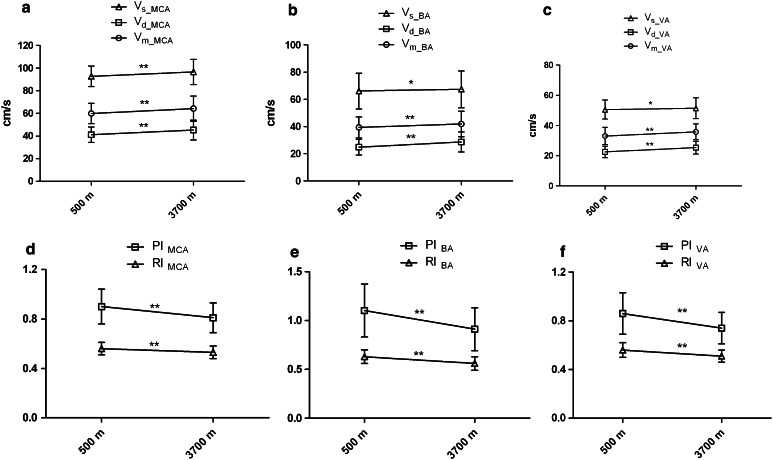
The alterations of cerebral hemodynamics from 500 to 3,700 m. The means of systolic velocity, diastolic velocity and mean velocity in bilateral VAs and MCAs (Fig. 1a, c), systolic velocity, diastolic velocity and mean velocity of BA (Fig. 1b), means of PI and RI in bilateral VAs and MCAs (Fig. 1d, f), PI and RI of BA (Fig. 1e). *p is 0.05 or less; **p is 0.01 or less

**Fig. 2:**
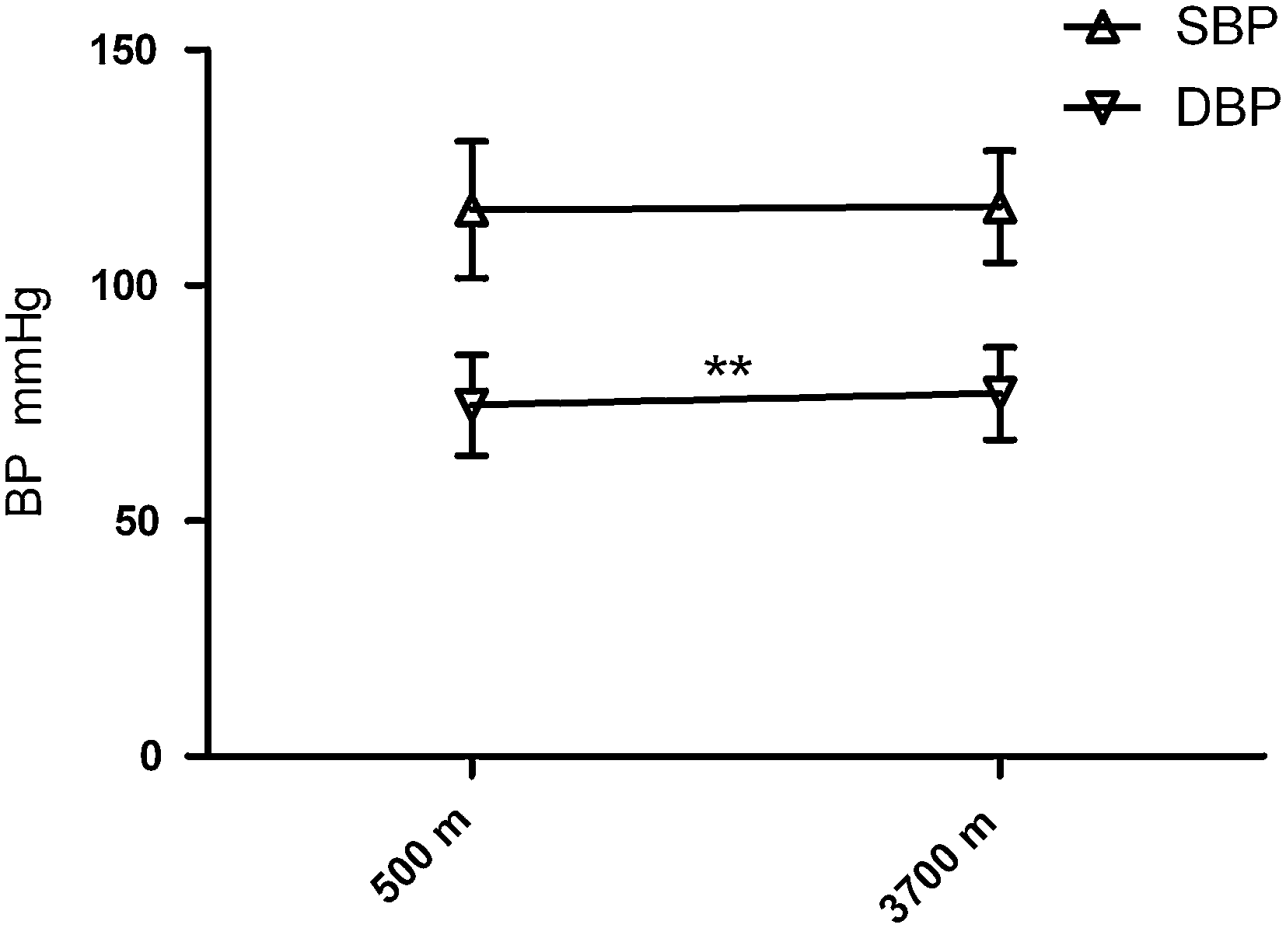
The alterations of BP from 500 to 3,700 m. *p is 0.05 or less; **p is 0.01 or less

**Fig. 3:**
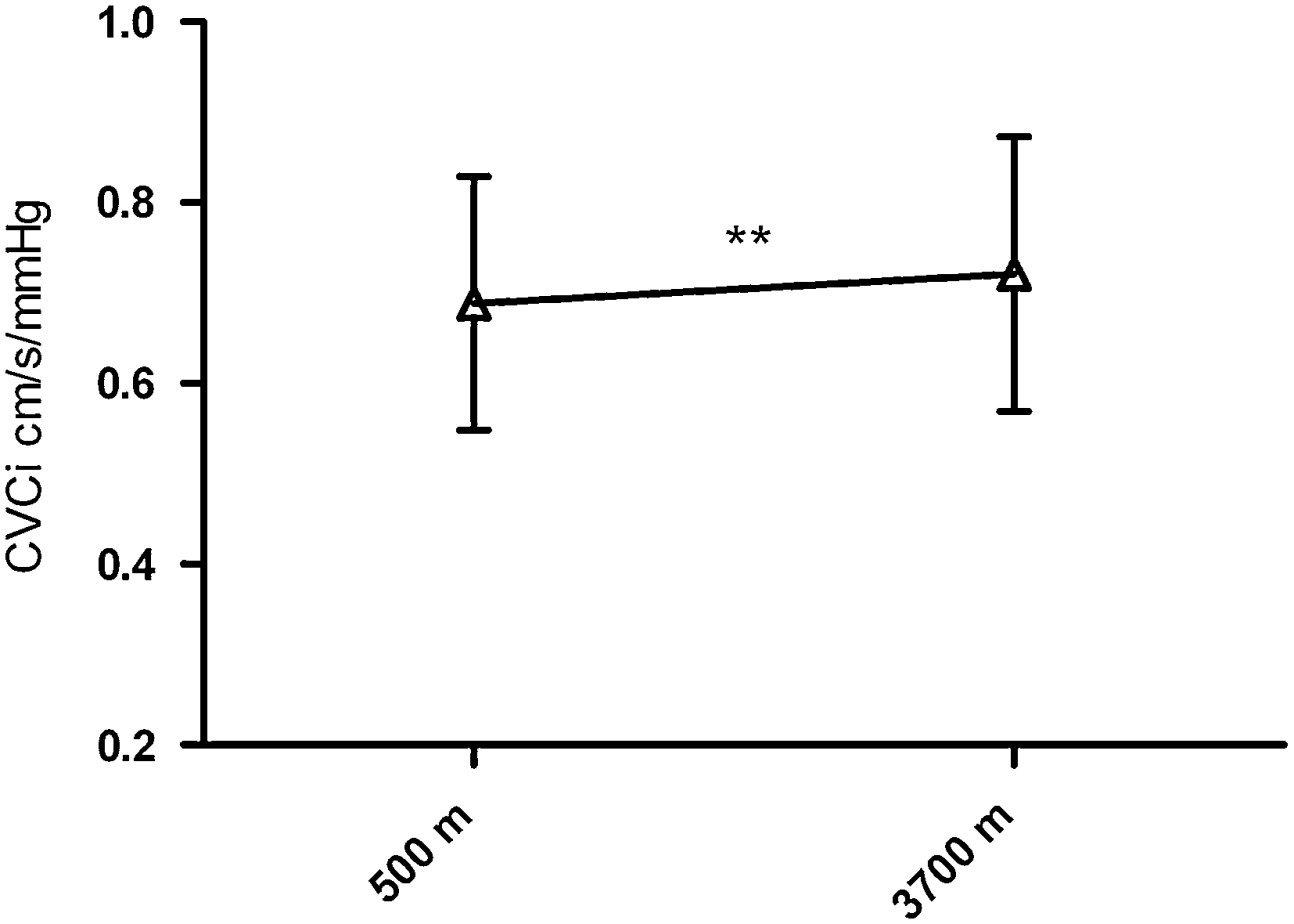
Change in cerebrovascular conductance index from 500 to 3,700 m. **p is 0.01 or less

The velocities of MCA, BA, and VA increased greatly from 500 to 3,700 m (all of the *p* values were less than 0.05) (Fig. 1a–c), while PIs and the RIs in these arteries decreased sharply (all of the *p* values were less than 0.01) (Fig. 1d–f). However, AI of MCA and VA was not significantly different between 500 and 3,700 m (*p* = 0.323 and 0.359, respectively). The DBP increased significantly (74 ± 11 vs. 77 ± 10 mmHg, *p* = 0.007) while change of SBP was not statistically significance from 500 to 3,700 m (Fig. 2). The relationship of systemic blood pressure and cerebral pressure, cerebrovascular conductance index, had changed dramatically from sea level to high altitude (0.69 ± 0.14 vs. 0.72 ± 0. 15 cm/s/mmHg) (Fig. 3).

Analyses of relationships between the AMS scores and the changes in CBF data have been performed. There were no significant relationships between AMS score and the changes of CBF. However, only the change of *V*_d_VA_ was significantly different between AMS+ and AMS− groups [3.00 (5.50) vs. 2.00 (6.00) cm/s].

After acute exposure at 3,700 m, there were no significant differences in the cerebral hemodynamic parameters of the anterior circulation or the MCA between the AMS+ and AMS− groups (*V*_s_MCA_, *V*_d_MCA_, *V*_m_MCA_ and velocities differences of bilateral MCAs, all *p* > 0.05). However, in the cerebral posterior circulation, *V*_s_BA_, *V*_d_BA_ and *V*_m_BA_ were much higher in AMS+ individuals than AMS− subjects (*V*_s_BA_, 66 ± 12 vs. 69 ± 15 cm/s, *p* = 0.017; *V*_d_BA_, 29 ± 7 vs. 31 ± 8 cm/s, *p* = 0.012 and *V*_m_BA_, 42 ± 8 vs. 44 ± 10 cm/s, *p* = 0.008), although neither PI_BA_ nor RI_BA_ was significantly different (*p* = 0.245 and 0.299) (Table 1). The AMS+ subjects experienced significantly faster *V*_d_VA_ than the AMS− subjects (*p* = 0.013), but *V*_s_VA_ and *V*_m_VA_ were not significantly higher (*p* = 0.286 and 0.052). However, the systolic/diastolic function and resistance indicators, PI and RI of the VA were significantly lower in AMS+ group than that in AMS− group (both *p* values were 0.004). Furthermore, the variations of *V*_s_VA_, *V*_d_VA_, and *V*_m_VA_ in the AMS+ group were significantly different from those in the AMS− group (*p* = 0.012, 0.003 and 0.015, respectively) (Table 1). Furthermore neither SBP (118 ± 11 vs. 119 ± 12 mmHg, *p* = 0.458) nor DBP (78 ± 11 vs. 79 ± 10 mmHg, *p* = 0.562) were different between AMS+ and AMS− groups. Furthermore, the cerebrovascular conductance index was not different in AMS+ group from the AMS− one (0.72 ± 0.14 vs. 0.73 ± 0.15 cm/s/mmHg, *p* = 0.278).

**Table 1:** Differences in cerebral hemodynamic characteristics between different groups at 3,700 m

	AMS			HAH			Dizziness		
	AMS− (162)	AMS+ (274)	*p*	HAH− (112)	HAH+ (324)	*p*	Dizziness− (123)	Dizziness+ (313)	*p*
PI_MCA_	0.81 ± 0.13	0.80 ± 0.12	0.171	0.81 ± 0.14	0.80 ± 0.12	0.434	0.82 ± 0.13	0.80 ± 0.12	0.031*
RI_MCA_	0.53 ± 0.05	0.53 ± 0.04	0.297	0.53 ± 0.05	0.53 ± 0.04	0.764	0.54 ± 0.05	0.53 ± 0.05	0.047*
*V*_s_BA_	66 ± 12	69 ± 15	0.017*	66 ± 11	69 ± 15	0.069	67 ± 12	68 ± 14	0.224
*V*_d_BA_	29 ± 7	31 ± 8	0.012*	29 ± 7	30 ± 8	0.074	29 ± 7	30 ± 7	0.097
*V*_m_BA_	42 ± 9	44 ± 10	0.008**	42 ± 8	44 ± 10	0.029*	42 ± 9	44 ± 10	0.108
*V*_d_VA_	25 ± 4	26 ± 4	0.013*	26 ± 4	26 ± 4	0.185	25 ± 4	26 ± 4	0.042*
*V*_m_VA_	36 ± 5	37 ± 6	0.052	36 ± 5	37 ± 5	0.242	36 ± 5	37 ± 6	0.081
PI_VA_	0.74 ± 0.14	0.71 ± 0.12	0.004**	0.74 ± 0.15	0.71 ± 0.12	0.061	0.74 ± 0.15	0.71 ± 0.12	0.071
RI_VA_	0.51 ± 0.07	0.49 ± 0.05	0.004**	0.51 ± 0.08	0.50 ± 0.05	0.068	0.51 ± 0.07	0.50 ± 0.05	0.046*
Δ*V*_s_VA_	−1.00 (8.00)	0.00(10.00)	0.012*	−2.00(8.00)	0.00 (10.00)	0.022*	−2.00 (8.00)	0.00 (10.50)	0.001**
Δ*V*_d_VA_	−2.00 (7.00)	−1.00 (6.00)	0.003**	−3.00(6.00)	−1.00 (6.75)	0.004**	−2.00 (6.00)	−1.00 (6.00)	0.001**
Δ*V*_m_VA_	−2.00 (8.00)	−1.00 (7.00)	0.015*	−3.00(7.00)	−1.00 (6.75)	0.015*	−2.00 (7.00)	−1.00 (7.00)	0.003**
AI_VA_ %	−5.7 (21.0)	−2.5 (17.8)	0.016*	−6.9 (21.0)	−2.7 (18.1)	0.014*	−6.9 (18.5)	−2.4 (19.6)	0.003**

The velocities: cm/s

* *p* is 0.05 or less

** *p* is 0.01 or less

In the symptomatology of AMS, headache and dizziness are the most important symptoms in LLS. Here, we observed that *V*_m_BA_, and the velocities differences of bilateral VAs were significantly different between HAH+ group and HAH− one (*p* = 0.029, 0.022, 0.004 and 0.015, respectively) (Table 1). There were significant differences between the populations with or without dizziness in PI_MCA_ (*p* = 0.031), RI_MCA_ (*p* = 0.047), *V*_d_VA_ (*p* = 0.042), RI_VA_ (*p* = 0.046), difference of systolic velocity in bilateral VAs (*p* = 0.001), difference of diastolic velocity in bilateral VAs (*p* = 0.001), and difference of mean velocity in bilateral VAs (*p* = 0.003).

The Spearman’s correlation analyses revealed that AMS score closely was correlated with the hemodynamic parameters of BA (*V*_m_BA_: *r* = 0.122, *p* = 0.011; *V*_s_BA_: *r* = 0.069, *p* = 0.017; *V*_d_BA_: *r* = 0.150, *p* = 0.002; PI_BA_: *r* = −0.137, *p* = 0.004; RI_BA_: *r* = −0.146, *p* = 0.002). Additionally, there were significant correlations between the AMS score and *V*_m_VA_ (*r* = 0.119, *p* = 0.013), *V*_d_VA_ (*r* = 0.149, *p* = 0.002), PI_VA_ (*r* = −0.163, *p* = 0.001), RI_VA_ (*r* = −0.171, *p* < 0.001), difference of mean velocity between bilateral VAs (*r* = 0.171, *p* < 0.001), difference of diastolic velocity between bilateral VAs (*r* = 0.179, *p* < 0.001), difference of systolic velocity between bilateral VAs (*r* = 0.162, *p* < 0.001) and AI_VA_ (*r* = 0.170, *p* < 0.001) (Table 2). In addition, the severities of HAH and dizziness were also closely correlated with many cerebral hemodynamic parameters (Tables 3, 4).

**Table 2:** Relationships between AMS score and cerebral hemodynamic parameters at 3,700 m

	AMS score														
	MCA		BA					VA							
	PI_MCA_	RI_MCA_	*V*_m_BA_	*V*_s_BA_	*V*_d_BA_	PI_BA_	RI_BA_	*V*_m_VA_	*V*_d_VA_	PI_VA_	RI_VA_	Δ*V*_m_VA_	AI_VA_	Δ*V*_d_VA_	Δ*V*_s_VA_
Spearman r	−0.105	−0.107	0.122	0.069	0.150	−0.137	−0.146	0.119	0.149	−0.163	−0.171	0.171	0.170	0.179	0.162
*p* value	0.028	0.025	0.011	0.017	0.002	0.004	0.002	0.013	0.002	0.001	<0.001	<0.001	<0.001	<0.001	0.001

The variables which were closely related to AMS score had been listed above

**Table 3:** Relationships between dizziness and cerebral hemodynamic parameters at 3,700 m

	Dizziness										
	MCA		BA			VA					
	PI_MCA_	RI_MCA_	*V*_d_BA_	PI_BA_	RI_BA_	*V*_m_VA_	*V*_d_VA_	Δ*V*_m_VA_	AI_VA_	Δ*V*_d_VA_	Δ*V*_s_VA_
Spearman *r*	−0.104	−0.104	0.102	0.099	−0.015	0.097	0.103	0.134	0.120	0.138	0.151
*p* value	0.030	0.030	0.032	0.039	0.028	0.044	0.032	0.005	0.012	0.004	0.002

The variables which were closely related to extension of dizziness had been listed above

**Table 4:** Relationship between HAH and cerebral hemodynamic parameters at 3,700 m

	HAH									
	BA		VA							
	*V*_m_BA_	*V*_d_BA_	*V*_m_VA_	*V*_d_VA_	PI_VA_	RI_VA_	Δ*V*_m_VA_	AI_VA_	Δ *V*_d_VA_	Δ*V*_s_VA_
Spearman *r*	0.103	0.099	0.108	0.123	−0.013	−0.104	0.119	0.138	0.135	0.114
*p* value	0.032	0.039	0.025	0.010	0.031	0.030	0.029	0.004	0.005	0.017

The variables which were closely related to HAH severities had been listed above

## Discussion

Large alterations in cerebral hemodynamics caused by rapid ascent from 500 to 3,700 m were detected in our study. The blood flow measured in the cerebral anterior and posterior circulation was greatly increased, while the PIs and RIs were significantly reduced. Cerebral hemodynamic characteristics in AMS individuals significantly differed from those in non-AMS subjects. The Spearman’s analyses revealed that AMS score was strongly correlated with the changes in the cerebral posterior circulation.

### Alterations in cerebral hemodynamics after rapidly ascending to high altitude

Velocities in the MCAs increased immediately upon acute exposure to high altitude, which was the same as the results reported in several studies (Berre et al. 1999; Lucas et al. 2011; Rootwelt et al. 1986; Rudzinski et al. 2007). We also found that the velocities of BA and VAs, which constitute the posterior cerebral circulation, were significantly altered from 500 to 3,700 m, which is partially consistent with a previous study (Huang et al. 1987). It had been thought that CBF increased primarily due to the balance between hypoxic cerebral vasodilatation and hyperventilation-induced cerebral vasoconstriction (Ainslie and Duffin 2009). Furthermore, a sharp increase in cerebral hemodynamics was detected in the present study, which also reflected the rise in CBF when the corresponding arterial diameter was unchanged or dilated.

However, a novel result was demonstrated in our study. The indicators of compliance and resistance, PIs and RIs, exhibited a sharp reduction upon acute ascent to 3,700 m. This rarely reported observation indicated that the cerebral arteries were converted to a lower compliance and resistance state, which may be due to the hypoxic cerebral vasodilatation. Thus, the increased velocities of the cerebral hemodynamics combined with the decreased pulsation indicators theoretically induced the increase in CBF, which was consistent with the previous studies (Berre et al. 1999; Lucas et al. 2011; Wolff 2000).

### Cerebral hemodynamic characteristics of AMS

In the current study, we also found that the hemodynamic parameters in the anterior circulation were not significantly different between AMS+ group and AMS− group either at the sea level or high altitude (Baumgartner et al. 1999; Lassen 1992). However, the compliance and resistance indexes were closely correlated with the AMS score at 3,700 m. These results were consistent with the study by Baumgartner et al. (1994). In contrast, we did not detect differences in Δ*V*_m_MCA_, inconsistent with the previous study (Baumgartner et al. 1994). The inconsistency between our study and Baumgartner’s may be caused by the sample sizes, the exposure durations and altitudes. As shown in the section of “Results”, individuals with HAH or dizziness at 3,700 m exhibited no difference in MCA velocity from those without HAH or dizziness.

There are some other novel findings that the parameters of cerebral posterior circulation were correlated to AMS closely. With respect to the cerebral posterior circulation, the vertebrobasilar arteries, which were reflected by velocities of BA and VAs, were significantly altered between AMS+ group and AMS− one, and these alterations were closely associated with the AMS score at high altitude. This effect may have been due to the inclusion of headache and dizziness in the LLS, which depends on the function of the cerebellum and the posterior cerebrum, and is supplied with blood and oxygen by VAs and BA. These novel findings may provide novel avenues for the study, prevention and prediction of AMS. On the other hand, HAH in many AMS patients also displayed pulsatile bursts (75.3 %) and occipital lobe presentation (3.6 %) characteristics (Serrano-Duenas 2005), which is influenced by the posterior lobe of the cerebrum and may be induced by the greater hyperperfusion state of this brain region.

A previous study indicated that oxygen delivery did not fall significantly at high altitude as a result of increased CBF (Wolff 2000). However, we found that *V*_s_BA_, *V*_d_BA_, *V*_m_BA_ and *V*_d_VA_ were significantly different between the AMS+ group and the AMS− group at 3,700 m, demonstrating that the development of AMS, especially the symptoms HAH and dizziness, were caused by the increase in CBF and the hyperperfusion state rather than the increased rates of oxygen metabolism.

The close relationship between AMS score and the velocities in the vertebrobasilar arteries was primarily due to the differences and associations among the neurological systems of AMS, HAH and dizziness, which mainly depended on the parameters of BA and VAs.

### Asymmetry of the bilateral VAs in AMS

The asymmetry of the bilateral isonym cerebral arteries existed in the normal physiological state, but no difference was detected between AMS+ and AMS− groups. However, physiological asymmetry could become a critical or pathological irregularity due to amplification via hypoxia-induced vasodilatation and increased blood flow. AMS subjects experienced a higher blood flow in the right VA or a lower blood flow in the left VA, especially in diastolic velocity.

Because VAs supply 2/5 areas of the cerebrum and the cerebellum, the regional regulation of brain blood flow has been studied for its important roles in AMS (Ainslie et al. 2008), which may be caused by a slightly impaired auto-regulation of CBF upon acute elevation exposure at 3,700 m. Although CBF increase is not different between white matter and gray matter, irrespective of AMS susceptibility, the asymmetrical blood flow was still potential risk factor for AMS(Dyer et al. 2008), especially the primary symptom.

Furthermore, the asymmetry of the VAs closely correlated with the AMS score which had not been reported in the previous studies. The variance between isonym cerebral arteries may play a major role in AMS, but further studies are required focusing on the precise pathophysiological mechanisms of AMS.

Although the relationship between AMS and CBF was still in argument, the cerebral cortical tissue remains hypoxic as indicated by the unregulated hypoxia inducible factor-1 (HIF-1) which may be involved in early high altitude cerebral edema (Bailey et al. 2009; Zhou et al. 2008). The AMS is a kind of multifactorial disease. Thus the cerebral hemodynamic characteristics AMS may be one of the components originating the disease, as well as a manifestation of it.

### Limitations

Our study was restricted to young Chinese men, which could perhaps generate a bias due to age or gender. Thus, the study would be improved in our future studies. Another limitation is that one key modulator of cerebral perfusion, PaCO_2_, has not been measured in the present study. This could be designed for further mechanism studies.

## Conclusions

AMS is associated with sharp changes in the cerebral hemodynamics of the posterior circulation rather than anterior circulation. AMS is also characterized by higher blood flow but a lower resistance, hyper-perfusion state. The asymmetry of the bilateral VAs may play a major role in the development of AMS, which warrants further study.

## Abbreviations

AMS: Acute mountain sickness
AMS+: With AMS
AMS−: Non-AMS
BA: Basilar artery
BP: Blood pressure
CBF: Cerebral blood flow
HAH: High-altitude headache
LLS: Lake Louise scoring system
MCA: Middle cerebral artery
PI: Pulsatility index
PI_BA_: PI of BA
PI_MCA_: Mean of PI in bilateral MCAs
PI_VA_: Mean of the PI in bilateral VAs
RI: Resistance index
RI_BA_: RI of the BA
RI_VA_: Mean of the RI in bilateral VAs
RI_MCA_: Mean of RI in bilateral MCAs
TCD: Transcranial Doppler
VA: Vertebral artery
*V*_s_BA_: Systolic velocity of BA
*V*_d_BA_: Diastolic velocity of BA
*V*_m_BA_: Mean velocity of BA
*V*_s_MCA_: Mean of systolic velocity in bilateral MCAs
*V*_d_MCA_: Mean of diastolic velocity in bilateral MCAs
*V*_m_MCA_: Mean of mean velocity in bilateral MCAs
*V*_s_VA_: Mean of systolic velocity in bilateral VAs
*V*_d_VA_: Mean of diastolic velocity in bilateral VAs
*V*_m_VA_: Mean of mean velocity in bilateral VAs
Δ*V*_s_MCA_: Difference in systolic velocity between bilateral MCAs
Δ*V*_d_MCA_: Difference in diastolic velocity between bilateral MCAs
Δ*V*_m_MCA_: Difference in mean velocity between bilateral MCAs
Δ*V*_s_VA_: Difference in systolic velocity between bilateral VAs
Δ*V*_d_VA_: Difference in diastolic velocity between bilateral VAs
Δ*V*_m_BA_: Difference in mean velocity between bilateral VAs
